# Marine protected areas increase temporal stability of community structure, but not density or diversity, of tropical seagrass fish communities

**DOI:** 10.1371/journal.pone.0183999

**Published:** 2017-08-30

**Authors:** Elisa Alonso Aller, Narriman S. Jiddawi, Johan S. Eklöf

**Affiliations:** 1 Department of Ecology, Environment and Plant Sciences, Stockholm University, Stockholm, Sweden; 2 Institute of Marine Sciences, Dar es Salaam University, Zanzibar, Tanzania; Department of Agriculture and Water Resources, AUSTRALIA

## Abstract

Marine protected areas (MPAs) have been shown to increase long-term temporal stability of fish communities and enhance ecosystem resilience to anthropogenic disturbance. Yet, the potential ability of MPAs to buffer effects of environmental variability at shorter time scales remains widely unknown. In the tropics, the yearly monsoon cycle is a major natural force affecting marine organisms in tropical regions, and its timing and severity are predicted to change over the coming century, with potentially severe effects on marine organisms, ecosystems and ecosystem services. Here, we assessed the ability of MPAs to buffer effects of monsoon seasonality on seagrass-associated fish communities, using a field survey in two MPAs (no-take zones) and two unprotected (open-access) sites around Zanzibar (Tanzania). We assessed the temporal stability of fish density and community structure within and outside MPAs during three monsoon seasons in 2014–2015, and investigated several possible mechanisms that could regulate temporal stability. Our results show that MPAs did not affect fish density and diversity, but that juvenile fish densities were temporally more stable within MPAs. Second, fish community structure was more stable within MPAs for juvenile and adult fish, but not for subadult fish or the total fish community. Third, the observed effects may be due to a combination of direct and indirect (seagrass-mediated) effects of seasonality and, potentially, fluctuating fishing pressure outside MPAs. In summary, these MPAs may not have the ability to enhance fish density and diversity and to buffer effects of monsoon seasonality on the whole fish community. However, they may increase the temporal stability of certain groups, such as juvenile fish. Consequently, our results question whether MPAs play a general role in the maintenance of biodiversity and ecosystem functioning under changing environmental conditions in tropical seagrass fish communities.

## Introduction

Marine protected areas (MPAs) have become one of the key marine conservation strategies worldwide. Their ability to rebuild fish stocks, increase local biodiversity, and strengthen ecosystem functions is well documented from various habitats [1–3]. However, a number of studies have questioned the generality of MPA effects, particularly in less well-studied ecosystems like soft-bottom seagrass beds [3,4]. For example, Quiros et al. [4] recently found that, in the Philippines, MPAs have weak or no effects on seagrasses after accounting for the influence of land use. In addition, no-take MPAs that exclude fisheries have often resulted in conflicts with resource users [5]. Consequently, there is a clear need to assess the effectiveness of MPAs, particularly in less well-studied habitats and geographical areas.

Recently, there has been an increasing interest in the potential ability of MPAs to buffer ecosystems from environmental change and temporal disturbances, such as extreme weather events [6,7] and outbreaks of consumers like sea urchins and crown-of-thorn starfish [8,9], thereby increasing ecosystem temporal stability. One of the mechanisms through which MPAs could increase stability is by increasing biodiversity (e.g. species richness [10–12]). Diverse communities are more likely to contain species that respond differently to environmental changes, due to different environmental tolerances or reduced competition [10,13,14]. A high diversity of responses to a shared disturbance may lead to compensatory changes (‘negative-covariance effect’) and an average stable community response (‘averaging effect’) [10]. Enhanced stability in protected areas has been described in relation to disturbance effects [9], over long-term trends [15–17], as well as through modelling experiments [18], suggesting that MPAs may enhance ecosystem resilience to impacts of climate change.

So far, few studies have assessed the potential ability of MPAs to buffer effects of environmental variability at shorter time scales (e.g. in terms of reducing effects of seasonal variability). In the Mediterranean, MPAs have been shown to decrease seasonal variability in fish density, biomass and diversity [19–21]. This ‘buffer effect’ was attributed to higher predation in MPAs, reducing recruitment fluctuations [19], and/or seasonal changes in human activities outside MPAs (e.g. fishing pressure [20,21]). It is also theoretically possible that higher diversity in MPAs could increase stability at shorter time scales, if different species respond differently to seasonal changes. Consequently, a combination of stabilising effects due to increased diversity in MPAs, and increased fluctuations in open-access areas due to variable human disturbances, could lead to higher temporal stability within MPAs.

In tropical regions, the yearly monsoon cycle is a major natural force that affects fish reproduction, recruitment [22,23], abundance and distribution [22,24–26]. Recent climate models predict that within this century the monsoon will exhibit a delayed onset and an earlier recession, together with intensified precipitation [27–29]. At the same time, extreme weather events and heat waves are expected to increase in frequency and duration [28,30]. Changes in the timing and severity of such meteorological conditions are likely to affect coastal fish communities and benthic habitats, with potentially severe impacts on ecosystem functioning, as well as fisheries [31–33]. Consequently, there is a pertinent need for more knowledge about the extent to which monsoon seasonality affects tropical fish communities and fish habitats, and the potential ability of MPAs to buffer those seasonal effects, to advance future management of ecosystems and the natural resources that humans depend upon.

Another factor strongly affecting coastal fish communities in shallow areas is the distribution and abundance of foundation species such as reef-building corals, seagrasses and perennial macroalgae. Seagrasses are marine flowering plants that form important recruitment and foraging habitats (‘seagrass beds’) for stationary and migrating fish on a global scale [34,35]. In both tropical and temperate regions, seagrass structural complexity (e.g. cover, shoot density, height) can strongly affect associated fish communities [36–38]. As the growth, and therefore cover and distribution of seagrasses, often changes with season [39–41], seasonality can indirectly affect fish communities through changes in habitat extent and quality [42]. Even though the potential indirect effects from natural and anthropogenic variability of foundation species could be as important as direct effects, indirect effects have been much less studied [43] (but see [44]). Moreover, the extent to which MPAs could buffer such indirect effects and increase ecosystem stability remains an open question.

In this study, we investigated whether MPAs can buffer direct and indirect (habitat-mediated) effects of monsoon seasonality on tropical fish communities associated with seagrass beds. We performed a field survey in two MPAs (no-take zones) and two unprotected (open-access) sites around Zanzibar (Tanzania), during three monsoon seasons in 2014–15. We hypothesized that: i) fish communities would be temporally more stable within MPAs than open-access sites in terms of fish density, diversity and community structure; ii) the indirect effects of seasonality (those mediated by changes in the seagrass habitat) would be at least as important as the direct effects; and iii) a higher fish community stability over time within MPAs could be explained by the stabilising effects of protected areas due to increased diversity. We tested these hypotheses for the overall fish community, as well as for different age classes: juveniles, subadults and adults.

## Methods

### Survey design

#### Ethics statement

All fieldwork and data collection was observational and non-extractive, and did not involve endangered or protected species. Consequently, there was no need for ethics approval. Research permits covering all aspects of the study were granted by the Zanzibar Research Committee, Revolutionary Government of Zanzibar. In addition, permissions to carry out the study in the two MPAs were granted by the management offices of the two MPAs, Chumbe Island Coral Park and Mnemba Island Marine Conservation Area.

#### Study sites

**Fig 1:**
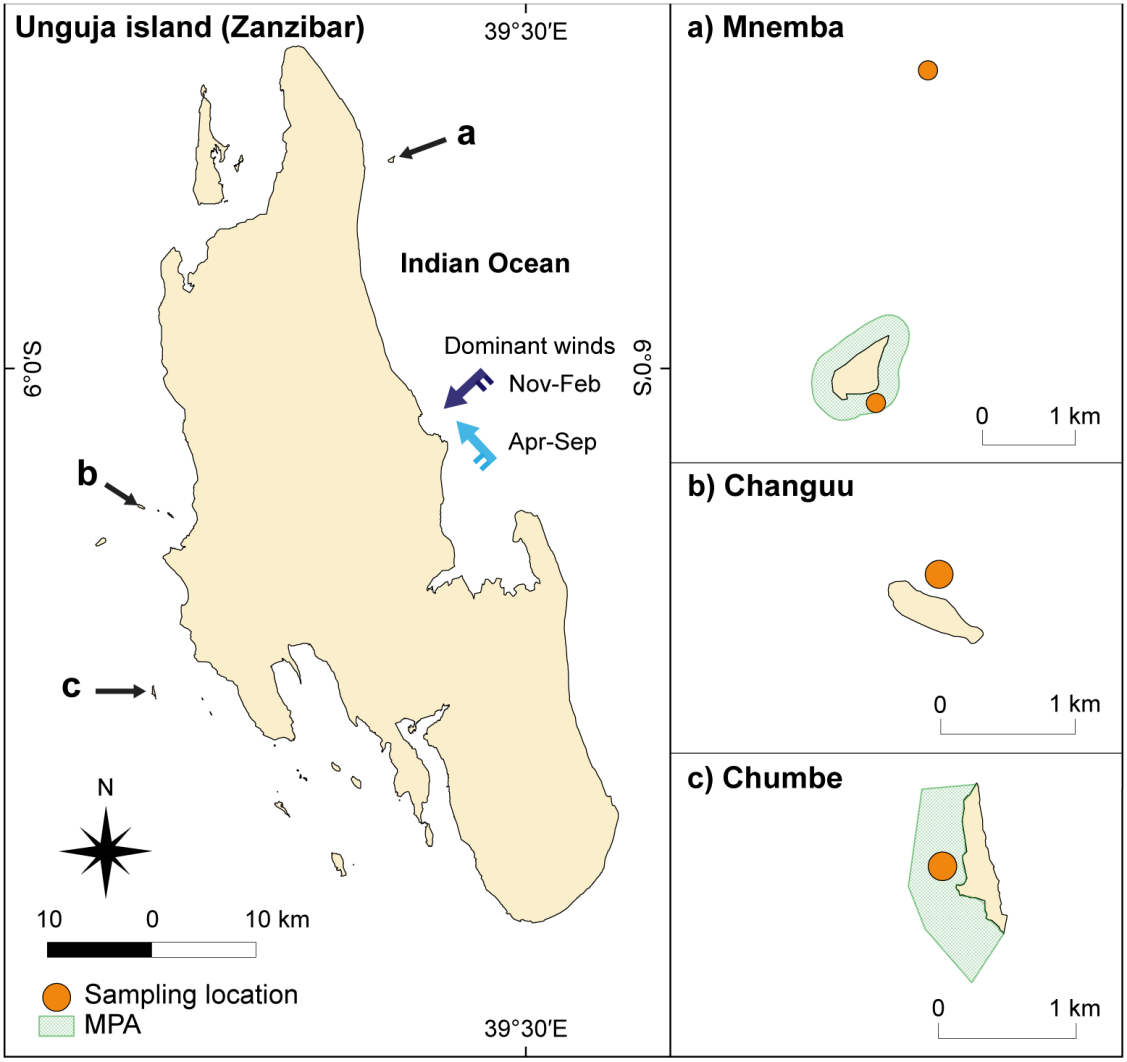
Map of Unguja Island, Zanzibar (Tanzania) and sampling locations. (A) Mnemba Island. (B) Changuu Island. (C) Chumbe Island. The green areas depict the MPAs, and the orange circles mark the four sampling sites. The blue arrows in the main map indicate the seasonal winds: dark blue is the north-easterly winds (Nov-Feb) and light blue the south-easterly winds (Apr-Sep). Background maps obtained from OpenStreetMap contributors (2016), available under the Open Database License (ODbL) at www.openstreetmap.org.

We conducted a field survey in four sites around Unguja Island (6.1°S 39.3°E) in the Zanzibar archipelago, Tanzania: two no-take MPAs (Chumbe and Mnemba Islands), each paired with an open-access (fished) area (Changuu island and Mnemba reef, respectively; Fig 1). Chumbe Island Coral Park (CHICOP) is a 0.3 km^2^, privately-run marine reserve that has been fully protected from fishing and other extractive activities since 1994 [45]. Mnemba Island is a private resort, where the sea area from 0–200 m from the shore has been formally protected from fishing since 2002, and is now part of the Mnemba Island Marine Conservation Area (MIMCA) [46]. These two MPAs are currently the only no-take zones in Unguja Island. The reference (open-access) sites were chosen based on their similarity to their respective MPA sites in terms of location, wave exposure and distance to coral reefs (see S1 Appendix), factors known to affect seagrass-associated fish assemblages [37,38,47,48].

#### Weather seasonality

**Fig 2:**
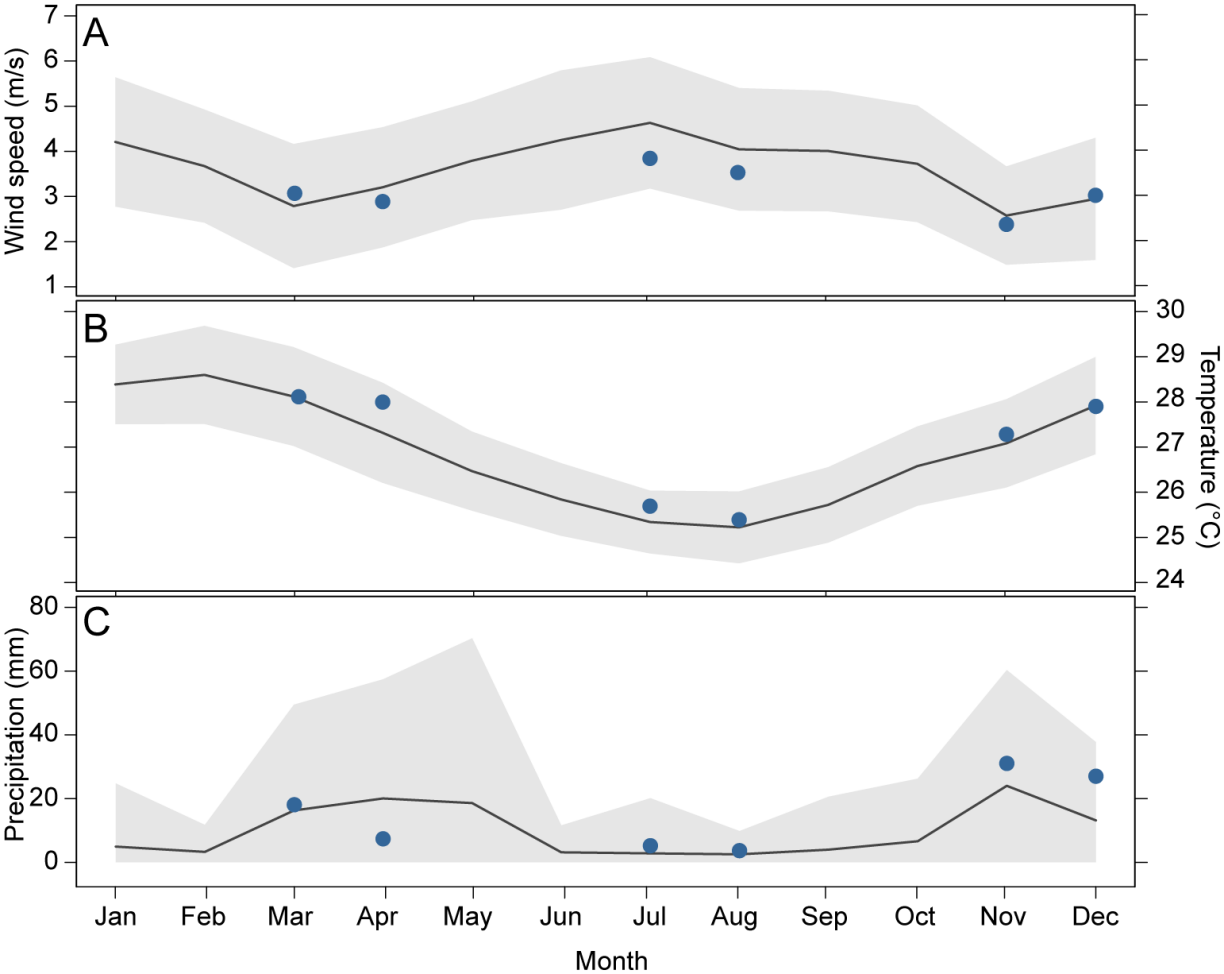
Seasonal variability of climate variables in Unguja Island, Zanzibar (Tanzania). (A) Average monthly wind speed (m/s). (B) Average monthly air temperature (°C). (C) Average daily precipitation (mm). Grey areas represent the standard deviations. Black lines represent average values for the period 2000–2015. Blue points represent the average monthly values during the sampling period of the present study: short rains (Nov-Dec 2014), long rains (Mar-Apr 2015), and dry season (Jul-Aug 2015). Climate data were obtained from NOAA/NCDC, available at http://gis.ncdc.noaa.gov/maps.

The monsoon cycle in the East African coastal region is characterised by two dry seasons (in January-February and April-September, respectively) and two rainy seasons (the long rains in March-May and the short rains in October-December) [22]. These exhibit distinct differences in temperature and rainfall (Fig 2). Winds also vary seasonally, in terms of both speed (Fig 2) and direction (Fig 1), with stronger south-easterly winds from April to September, and weaker north-easterly winds from November to February [22].

#### Artisanal fisheries

In the East African region, and particularly in Zanzibar (Tanzania), most fisheries are small-scale and artisanal, and operate from traditional vessels in nearshore waters [49,50]. The fisheries operate throughout the coastline in shallow-water habitats, with seagrass beds being among the most important fishing grounds [50,51]. A wide variety of fishing gears are commonly used (e.g. beach seine nets, basket traps, spear guns, hook and line), making fisheries in this region highly unselective, with almost any available species being targeted and caught, regardless of size [50,52]. Fishing activities vary over the year with the monsoonal patterns, both in terms of fishing pressure and fishing gears used [22,50,52] (but see [53]). Specifically, fishing pressure is lower between June and September, since strong winds and high waves limit the access to exposed sites during this time [50,54].

#### Field sampling

The study was conducted during three seasons in 2014–2015: short rains (November-December 2014), long rains (March-April 2015) and dry season (July-August 2015). Sampling was carried out in sub-tidal seagrass beds dominated by *Thalassodendron ciliatum* Forskål with >50% bottom cover. This tropical seagrass, which has leaf clusters growing from an unbranched or slightly branched stem, forms large monospecific and multispecies seagrass beds that harbour diverse and abundant fish communities [55].

At each site, fish and seagrass sampling was conducted using 10 replicate transects (except in Chumbe, where only 7 transects could be sampled due to the limited size of the protected seagrass bed), which were randomly placed > 5 m apart and parallel to the shore. Sampling was replicated during the three seasons, yielding a total of 111 transects [(10 + 10 + 10 + 7) × 3]. The surveys were carried out during high neap tide (± 3 h) through underwater visual census by snorkelling along 25×4 m belt transects, which is a standard method in tropical coral reef [56–58] and seagrass [38,59–61] fish surveys. Like all fish survey methods, visual census has its sources of error (e.g. underestimation of fish densities, overestimation of size, failure to detect cryptic species, diver effects on fish behaviour). However, as the method has been frequently used in the past, and to be able to compare our results with other studies, we used it here as well. After deploying a 25 m long transect line on the bottom, a waiting period of 5–10 min was allocated to reduce disturbance on fish. A snorkeler (always E Alonso Aller) then swam the transect twice at a constant swimming speed (~0.1 m/s), first identifying and counting all mobile and large fish, and second, the less mobile and small fish. All fish observed within or crossing the transect were identified to the lowest taxonomic level possible (usually species), counted, and their body length was visually estimated to the nearest 5 cm [59,61]. Based on the estimated sizes, all individual fish were then grouped into three age classes (juveniles, subadults and adults; following [62]). The bottom per cent cover of seagrass and other habitat-forming, sessile organisms (e.g. corals, macroalgae, sponges) were visually estimated (to the nearest 10%) within 0.25 m^2^ quadrates placed at 5 m intervals along each transect (resulting in six subsamples per transect, which were averaged before statistical analyses).

### Statistical analyses

All statistical analyses were conducted using R v. 3.3.1. [63]. Response variables were checked for normality and homogeneity of variance before analyses. For all analyses, significance levels were set at α = 0.05.

#### Effects of management and season on fish density and diversity

We used linear mixed effects models to evaluate the individual and interactive effects of i) season (three levels: short rains; long rains; and dry) and ii) management (two levels: MPA and open-access) on fish density and diversity. We have previously shown that variability in seagrass cover can influence fish assemblages [38], and should ideally be included as a covariate. However, when seagrass cover was included in the models, it resulted in multicollinearity (assessed using the Variance Inflation Factor). Therefore, we excluded this covariate from these analyses, and instead used piece-wise structural equation modelling (see below) to tease part the influence of seagrass cover.

Fish density was calculated as number of i) total individuals (pooling all size classes and species), ii) juveniles, iii) subadults and iv) adults per transect (100 m^2^). Species diversity per transect was calculated as species richness (*S*, number of species) and Shannon Index (*H’*, based on the relative abundances of the different species) using the {vegan} R package [64]. Site (four levels) was included as a random factor (random intercept only) nested within each level of management to account for the natural variability between sites [65]. Prior to analyses, fish densities were square root (√) transformed to obtain normal distribution, and all data were standardized (by subtracting the mean and dividing by the standard deviation) to allow for comparison of coefficients. Linear mixed effect models were fitted with the ‘lmer’ function from the {lme4} R package [66]. The validity of mixed effect models was evaluated by likelihood ratio tests, comparing the models to the null models with only random effects. R^2^ values of the models were obtained using the ‘r.squaredGLMM’ function from the {MuMIn} R package [67]. When a significant interaction between factors was included, we assessed the interaction through Tukey’s all-pair comparisons (‘lsmeans’ in {lsmeans} [68]). Tukey’s all-pair comparisons of means were also used when season was a significant predictor in the model to assess significance of differences between all three levels within the factor. Each transect was used as a replicate (N = 111).

#### Effects of management and season on fish community structure

To explore the single and joint effects of season and management on the multivariate fish assemblage structure (based on abundances of individual species), we first ran an unconstrained permutational multivariate analysis of variance (PERMANOVA) with the ‘adonis’ function from the {vegan} R package [64] with season, management and an interaction between the two factors as predictors of the variability in the community structure. The ‘adonis’ analysis also allowed us to account for the nestedness of our data, by including the factor site (nested within management levels) as strata, within which permutations were constrained. This analysis was based on Bray-Curtis dissimilarities and 999 permutations. We then fitted a constrained analysis of principal coordinates (CAP) to visualize the results. The CAP analysis was run with the ‘capscale’ function from the {vegan} R package [64] based on Bray-Curtis dissimilarities and 999 permutations. Finally, we ran a Similarity Percentages analysis (SIMPER) to evaluate which species were responsible for the observed differences (when present). SIMPER analyses were run with the ‘simper’ function from the {vegan} R package [64] based on 999 permutations. For these analyses we used site-level community data, based on the summed abundance of each species over all transects at each study site and season (resulting in 1 density value per species, site and season).

Second, we compared the temporal stability in community structure in MPAs and open-access sites. We applied nMDS based on Bray-Curtis dissimilarities (calculated using the {vegan} R package) [64] and used both multivariate distance- and area-based metrics of ordination scores to compare the magnitude of temporal dissimilarity in site-level community structure between MPAs and open-access sites (see [9] for example). We included four previously used metrics of temporal community variability (the inverse of stability): area, range, mean distance to centroid, and mean distance between points [9,69,70]. Area is the area of the polygon delimited by dots, which indicates the total extent of the community structure over all three seasons. Range is the maximum distance between dots, indicating the most extreme change in community structure. Mean distance to centroid is the average distance between each dot and its polygon centroid, and is a measure of the departure from the average community structure. Mean distance between points is the average distance between each dot and the previous one, indicating the mean community dissimilarity between seasons. In cases when area and range may be affected by outliers (one extreme dot), mean distance to centroid and mean distance between points may be better measurements of the overall variability at each site. We then estimated the effects of MPAs (vs. open-access sites; fixed factor) on each of these metrics using linear mixed effects models (‘lmer’ in {lme4}), including site as a random factor (nested within management levels). This procedure was performed to estimate the temporal variability of the total fish community, as well as the different age classes (juveniles, subadults, and adults).

#### Mechanisms affecting fish community stability

Since seagrass cover may vary between seasons [39,40], and may in turn affect local fish communities [38], we investigated the direct and indirect (seagrass cover-mediated) effects of seasonality and management on the fish community using piecewise structural equation modelling (SEM). SEM is a standard methodology used to tease apart net relationships into direct and indirect (mediated) relationships, based on previous systems knowledge of causal links between variables and observed data [71]. Piecewise SEM is an extension of SEM that overcomes some the restrictions of classical SEM models, for instance by accounting for non-independence of data by including random variation [72]. We first fitted general models for each univariate response variable (total fish density, juvenile, subadult and adult fish densities, and species richness and Shannon index), including an interaction between season and management (see S2 Appendix). When the interaction had a significant effect, we ran individual models for each level of management (MPAs vs. open-access sites) to understand the interaction. Models were fitted with the {piecewiseSEM} R package [72]. Each component of the models was fitted as a linear mixed effects model, with site as a random factor. The variable season, being a factor with 3 levels, was modelled as a composite variable [73], and thus the sign of coefficients for this variable cannot be interpreted. Each transect was used as a replicate (MPAs models: N = 51; open-access sites models: N = 60). In the results section, only models with Fisher’s C statistic P > 0.05 were selected, meaning that the models represented the data well and that there were no missing paths.

Second, we investigated the pair-wise correlations between densities of different fish species, both within MPAs and within open-access sites. If increased temporal stability within protected sites is explained by the diversity-stability hypothesis, we would expect MPAs to exhibit more negative correlations (negative-covariance effect) and/or an average neutral correlation between species (averaging effect). At the same time, if lower stability in open-access sites is explained by seasonal fluctuations in fishing pressure (which affects most species in the same way), we would expect open-access sites to display more positive correlations. Consequently, we calculated the slopes of correlation between the densities of species pairs per level of management (MPAs and open-access sites separately). This was done on site-level data (1 mean density value per species, site and season) and only for species found in both MPAs and open-access sites, to avoid species-specific differences confounding the comparison. We then ran linear mixed effects models (‘lmer’ in {lme4}) with site as a random factor for each pair of species for each level of management (MPAs and open-access sites). Finally, we compared the mean slopes of species correlations between MPAs and open-access sites using a two-sample paired t-test (‘t.test’ in {stats}) [63].

## Results

### General characteristics of the fish assemblages

Across the four sites and three seasons, we identified and estimated the length of 5696 individual fish. These belonged to 116 taxa (114 identified to species level) from 33 different families, with the five most common (corresponding to 80% of all individuals) being Scaridae (25%), Pomacentridae (19%), Labridae (17%), Acanthuridae (11%) and Siganidae (9%).

**Fig 3:**
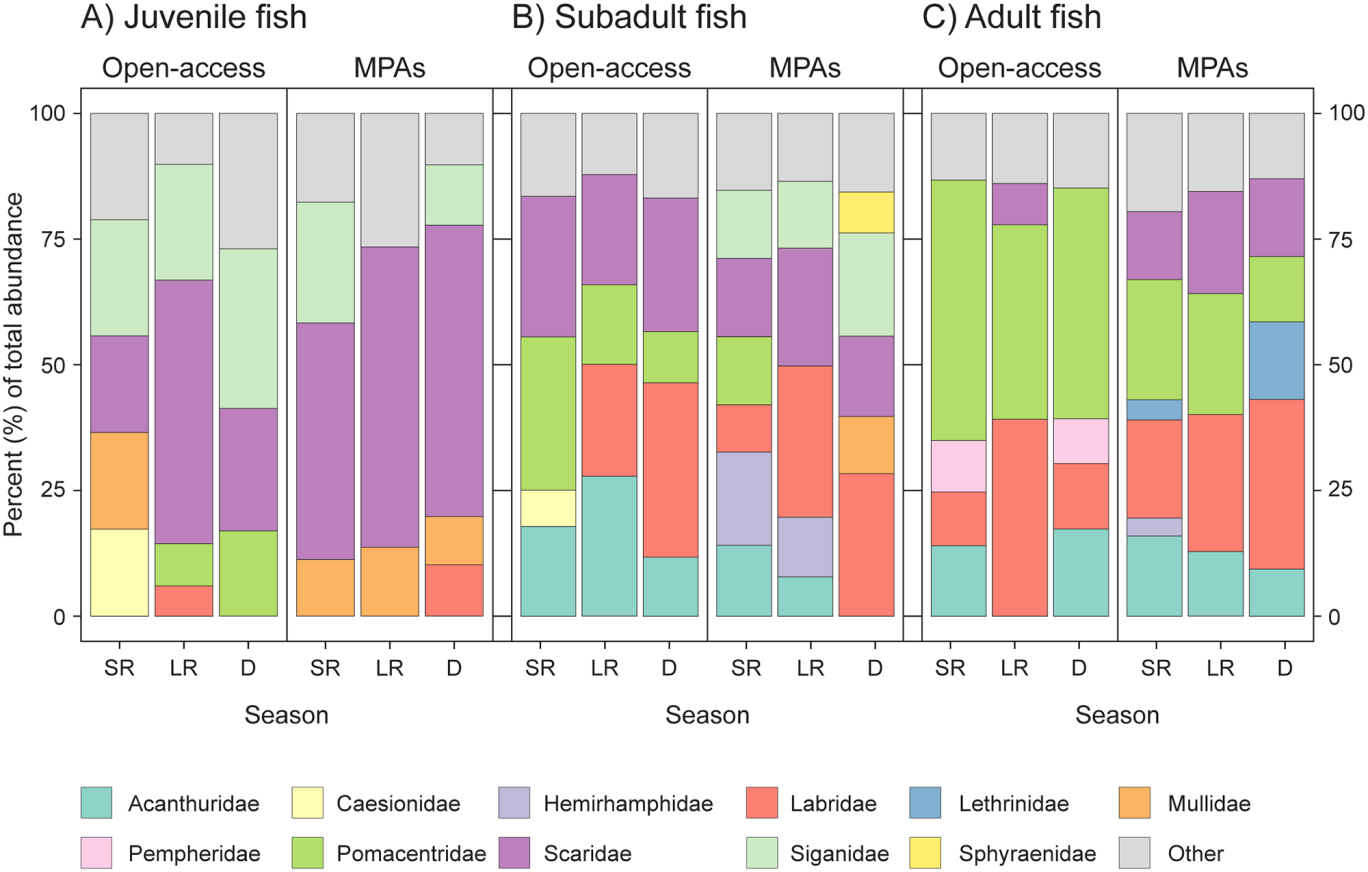
Community structure per season and management level based on fish families. Percentage number of (A) juvenile, (B) subadult, and (C) adult fish belonging to each family in MPAs and open-access areas during each season.

In terms of age classes, subadults were slightly more common (40%) than adults (32%) and juveniles (28%). Within MPAs, the juvenile fish community was dominated by species from the family Scaridae (47 to 59%) throughout all seasons, and Siganidae during the short rains and dry season (24 and 12%) (Fig 3A). In open-access sites, the dominant species varied more with the seasons, with Siganidae being abundant during all three seasons (23 to 31%), while Mullidae was only abundant during the short rains (19%), and Scaridae during the long rains and dry season (52 and 24%) (Fig 3A). Regarding the subadult fish community, in open-access sites some of the most common families among subadult fish were Pomacentridae, during the short rains (30%); Scaridae, during the short rains and dry season (27%); Acanthuridae, during the long rains (27%); and Labridae, during the long rains and the dry season (22 and 35%) (Fig 3B). In the MPAs, no family clearly dominated the subadult fish community during the short rains, while Labridae was mostly abundant during the long rains and dry season (30 and 28%), Scaridae during the long rains (23%), and Siganidae during the dry season (20%) (Fig 3B). Among adult fish, Pomacentridae (38 to 52%) was the most common family in open-access sites throughout all three seasons, while Acanthuridae was common during the short rains and the rainy season (14 and 17%) and Labridae (39%) during the long rains. Within MPAs, the adult fish community was mainly composed by Labridae throughout all seasons (19 to 33%), Pomacentridae during the short and long rains (23 and 24%) and Lethrinidae (15%) and Scaridae (15%) during the dry season (Fig 3C).

**Fig 4:**
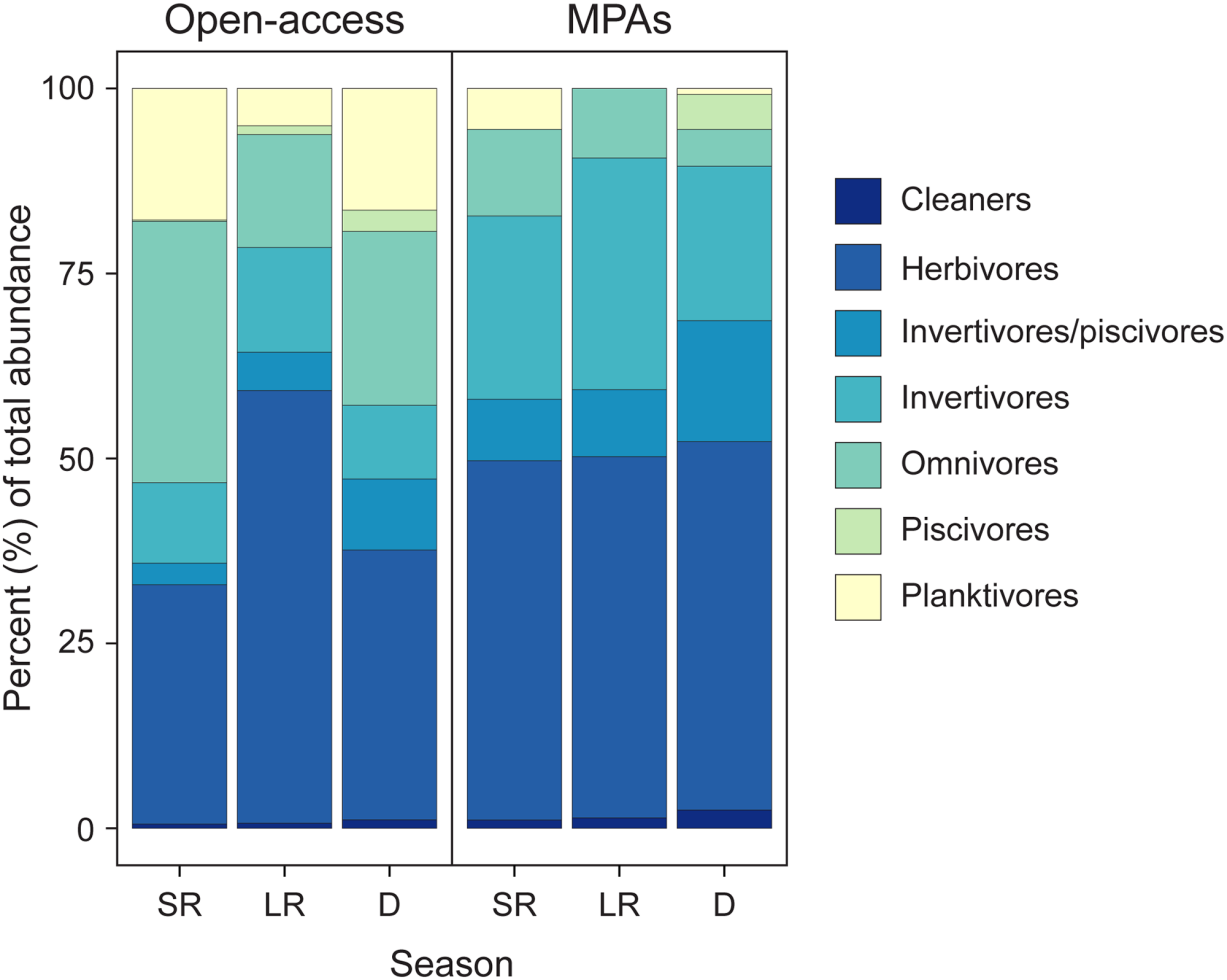
Community structure per season and management level based on functional groups. Percentage number of fish classified into functional groups based on feeding guilds in MPAs and open-access areas during each season.

Regarding functional groups, the fish assemblages were dominated by herbivores in both MPAs and open-access sites (30–40% of the total fish abundance at any given season), followed by invertivores within MPAs (25 to 35%) and omnivores in open-access sites (20 to 35%) (Fig 4).

### Effects of MPAs and season on fish density and diversity

**Fig 5:**
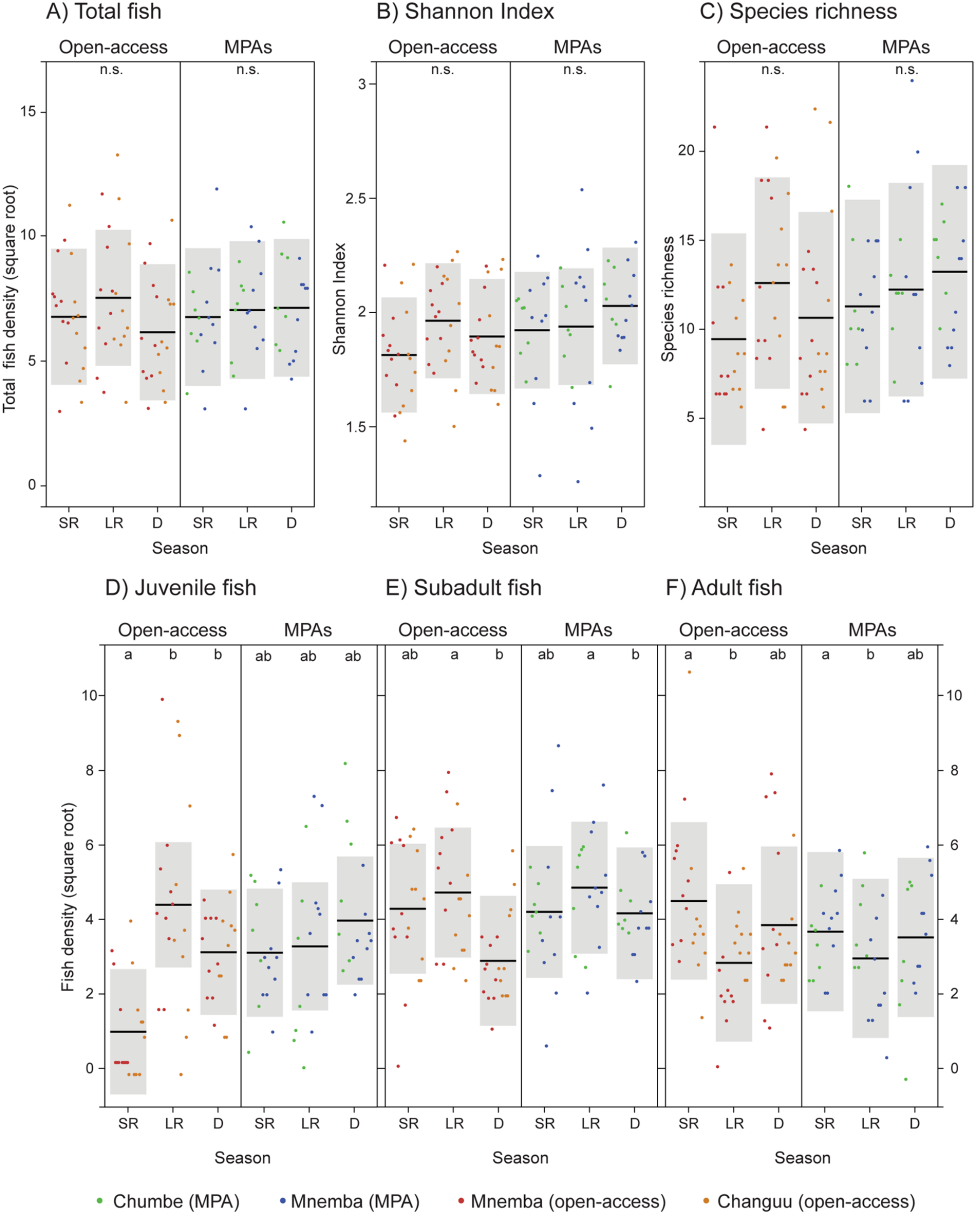
Effects of seasonality and management on fish densities. Conditional effect plots of linear mixed models for (A) total fish density (square root), (B) Shannon Index, (C) species richness, (D) juvenile fish (square root), (E) subadult fish (square root) and (F) adult fish density (square root), predicted by the interaction between management and season. Black lines represent the mean value and grey areas are 95% confidence intervals. Dots represent partial residuals (coloured by site, see figure legend). SR = short rains; LR = long rains; D = dry season. Significance is marked with letters (a and b) above each figure (n.s. indicates not significant).

Management and seasonality had no single or joint effects on total fish density, species richness or Shannon Index (Table 1, Figs A-C in Fig 5). Juvenile fish density, however, changed between seasons, and the season effect differed between protected and open-access sites (season × MPA interaction; Table 1). Tukey’s all-pair comparison of means showed that seasonality affected juvenile fish density in open-access sites but not within MPAs (Table 1, Fig 5D). Across the four sites, densities of subadults and adults differed between seasons, such that subadult fish density was higher during the long rainy season than during the dry period, and adult density was higher during the short rainy season than during the long rainy season (Table 1, Fig 5E and 5F). Management had no effect on subadult and adult fish densities. Although in the models for both subadult and adult fish densities most of the variance was explained by the random factor site (R^2^_conditional_ >> R^2^_marginal_), the models were significantly different (P = 0.005 and 0.006, respectively) from the null model (including only the random factor).

**Table 1:** Results of linear mixed effect models predicting fish densities (total, juvenile, subadult and adult fish) and species diversity (species richness and Shannon Index). Differences from Tukey’s all-pair comparison of means are shown for significant predictors.

Predictors		Coefficient	SE	*F*-statistic	DFnum	DFden	*P*-value	*R*^*2*^_*marginal*_	*R*^*2*^_*conditional*_
Total fish density								0.023	0.426
Management				0.007	1	2	0.933		
Season				1.047	2	103	0.351		
Season × Management				1.069	2	103	0.343		
Juvenile fish density								0.207	0.392
Management				0.309	1	2	0.579		
Season				10.549	2	103	< 0.001		
Season × Management				6.719	2	103	0.001		
Open-access	SR–LR	-1.499	0.265				< 0.001		
	SR–D	-0.938	0.265				0.001		
	LR–D	0.560	0.265				0.092		
MPA	SR–LR	-0.075	0.288				0.963		
	SR–D	-0.379	0.288				0.389		
	LR–D	-0.304	0.288				0.543		
Subadult fish density								0.102	0.418
Management				0.136	1	2	0.712		
Season × Management				2.002	2	103	0.135		
Season				6.519	2	103	0.001		
SR–LR		-0.293	0.197				0.302		
SR–D		0.387	0.197				0.126		
LR–D		0.680	0.197				0.002		
Adult fish density								0.064	0.465
Management				0.055	1	2	0.814		
Season × Management				0.748	2	103	0.473		
Season				5.261	2	103	0.005		
SR–LR		0.591	0.192				0.007		
SR–D		0.200	0.192				0.552		
LR–D		-0.391	0.192				0.108		
Shannon index								0.028	0.028
Management				0.476	1	2	0.490		
Season				0.063	2	103	0.939		
Season × Management				1.287	2	103	0.276		
Species richness								0.016	0.016
Management				0.443	1	2	0.506		
Season				0.077	2	103	0.927		
Season × Management				0.606	2	103	0.546		

SE = Standard error. DFnum = numerator degrees of freedom. DFden = denominator degrees of freedom. R^2^_marginal_ = variance explained by the fixed factors (here predictors). R^2^_conditional_ = variance explained by both fixed (predictors) and random factors (site). SR = Short Rains; LR = Long Rains; D = Dry season

### Effects of MPAs and season on fish community structure

**Fig 6:**
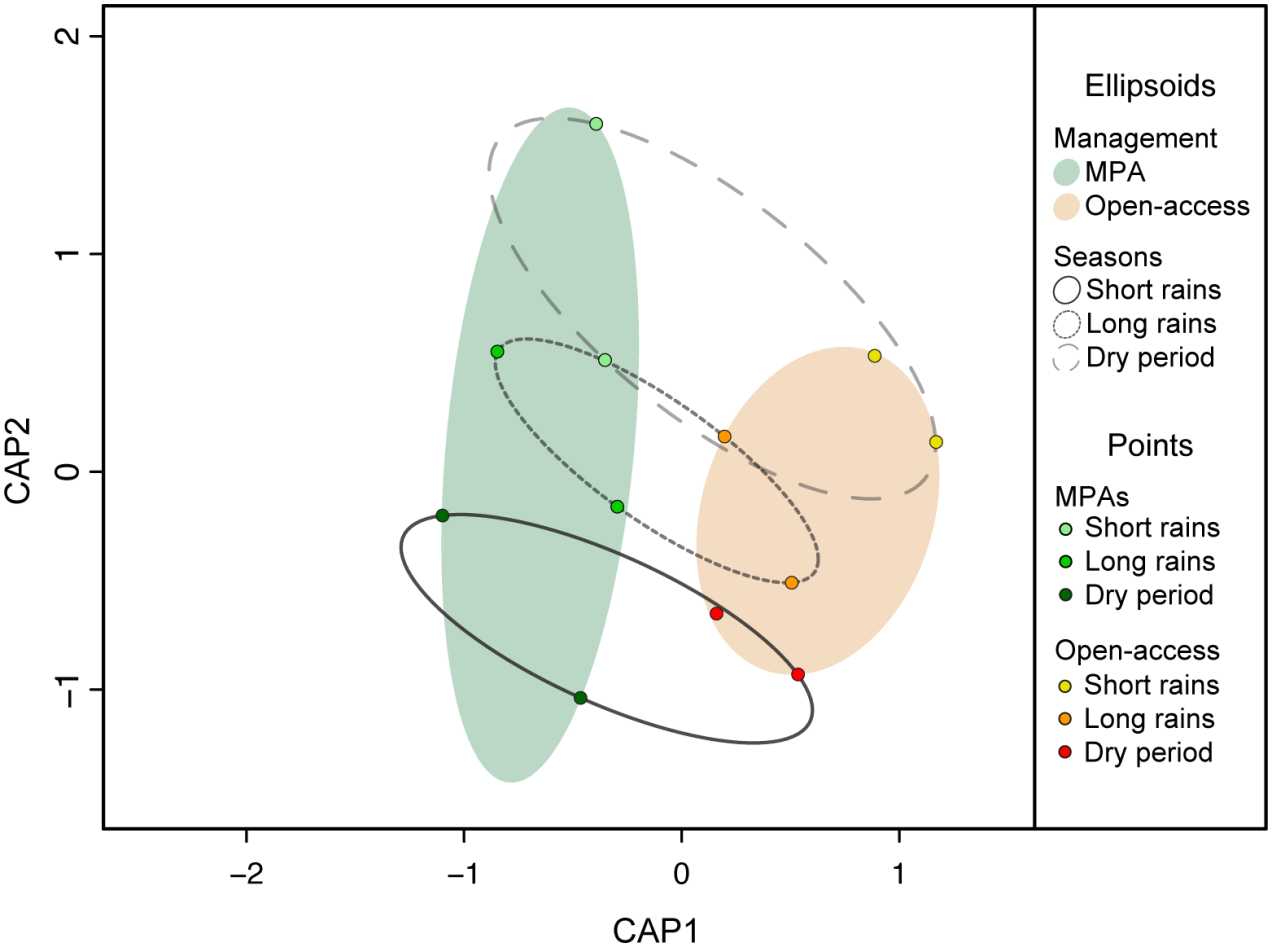
Effects of seasonality and management on fish community structure. Results from the CAP analysis with season and management as constraints. Ellipsoids represent the minimal area that encloses all points in the group. Ellipsoids in colour represent management level (MPAs: green; Open-access sites: orange), and the empty ellipsoids represent season (dashed, light grey: short rains; dotted, grey: long rains; solid, dark grey: dry season). Dots represent individual sites and are coloured by management level and season (see figure legend).

There was a significant effect of seasonality (F = 0.89, P = 0.005) and management (F = 1.54, P = 0.003) on total fish assemblage structure, although the interaction between the two factors was not significant (F = 0.55, P = 0.271). Consequently, assemblage structure differed between management levels and seasons (Fig 6), but the effect of seasonality was not influenced by protection. The differences between MPAs and open-access sites were mostly driven by the rabbitfish *Siganus sutor* and the parrotfish *Calotomus carolinus*, these being more abundant in MPAs compared to open-access sites, as well as a two species of damselfish (Pomacentridae) which were more abundant in open-access sites. The seasonal differences in assemblage structure were mostly driven by the parrotfish *Calotomus spinidens*, which was more abundant during the long and short rains, compared to the dry season, and the emperor *Lethrinus mahsena*, being more abundant during the long rains and dry season, compared to the short rains.

**Fig 7:**
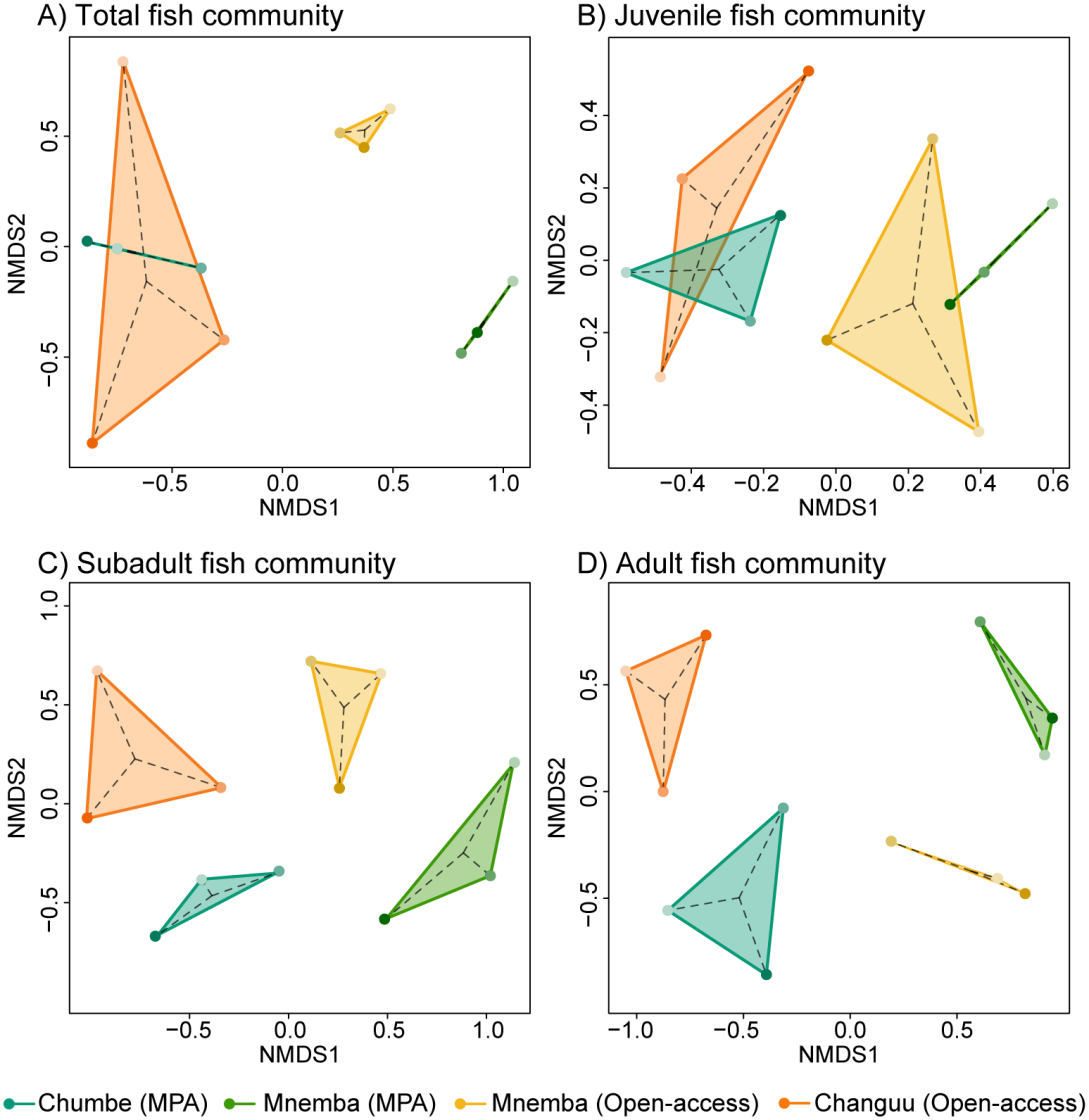
nMDS plots displaying community structure in MPA and open-access sites. (A) Total fish, (B) juveniles, (C) subadults, and (D) adult fish. Dots represent the community structure at each season, with seasons distinguished by shading (light: short rains; medium: long rains; dark: dry season). Each colour represents a particular site (see figure legend).

**Fig 8:**
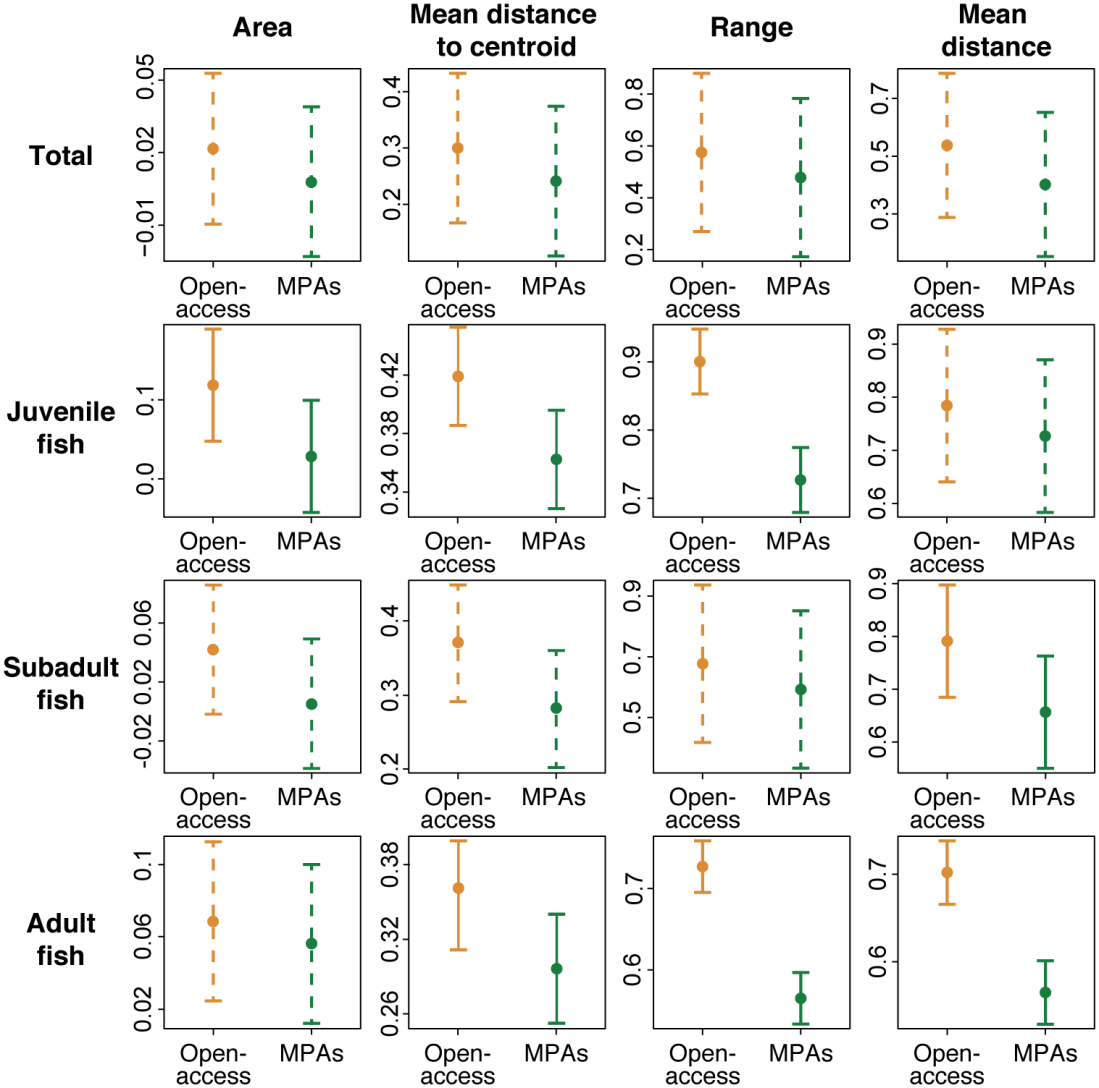
Metrics of temporal dissimilarity. Conditional effect plots of linear mixed models for the estimates of four different measures of temporal dissimilarity, extracted from the nMDS plots in Fig 7, predicted by level of management for total, juvenile, subadult and adult fish communities. Error bars indicate the 95% confidence intervals. Lines are coloured by management level (MPAs: green, Open-access sites: orange) Non-significant differences (P > 0.05) are represented by dashed lines.

The analyses of temporal stability in fish community structure (Fig 7) indicate that the temporal variability in community structure (the inverse of stability) was for certain age classes higher in open-access sites than within MPAs (Fig 8). Based on juvenile fish, the multidimensional area, range and mean distance to centroid were greater in open-access sites compared to MPAs (Fig 8), meaning that the juvenile assemblage in open-access sites underwent stronger compositional changes over the seasons. Based on subadult fish, the mean distance between points was greater in open-access than MPA sites (Fig 8), meaning that there was a greater between-seasons mean community dissimilarity in open-access sites. Based on adults, the mean distance to centroid, range, and mean distance between points were higher in open-access than in MPA sites (Fig 8), showing that open-access sites experienced larger changes in community structure. However, when considering the whole fish community, no significant differences were found for any of the metrics (Fig 8).

### Mechanisms regulating community stability

#### Direct vs. habitat-mediated effects of seasonality

**Fig 9:**
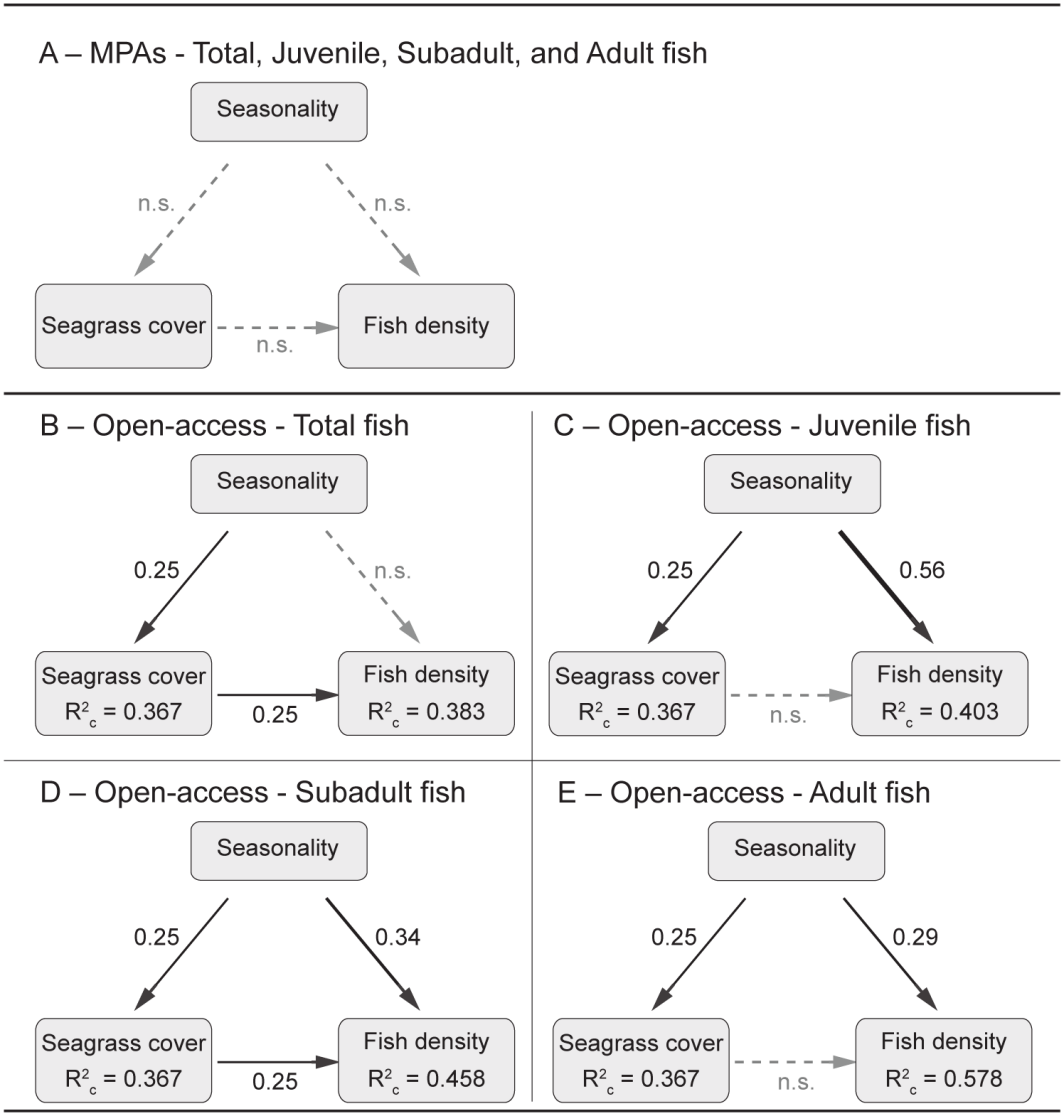
Direct and indirect (habitat-mediated) effects of seasonality on fish densities. Piecewise SEM exploring the effects of seasonality on fish densities in MPAs and open-access sites. (A) Model for total, juvenile, sub-adult and adult fish densities in MPAs; (B) model for total fish density in open-access sites; (C) models for juvenile fish density in open-access sites; (D) model for sub-adult fish density in open-access sites; and (E) model for adult fish density in open-access sites. Black arrows represent unidirectional relationship among variables (*P* ≤ 0.05), and their thickness has been scaled based on the magnitude of the standardized regression coefficient, given next to the arrows. Grey dashed arrows denote non-significant paths (*P* ≥ 0.05). R^2^ value for each component of the model is reported in the boxes of response variables as the conditional R^2^, which considers the effects of both fixed and random factors.

The results of the piecewise SEM analyses showed that there were both direct and indirect (habitat-mediated) effects of seasonality on fish, that depended on both protection and fish age class. Within MPAs there were no direct or indirect (seagrass-mediated) effects of season on fish densities within any of the four age classes (Fig 9A). In open-access sites, however, season had i) a direct effect on densities of juvenile and adult fish (Fig 9C and 9E); ii) both a direct and an indirect effect (mediated by changes in seagrass cover) on subadult fish density (Fig 9D); and iii) an indirect effect (mediated by seagrass cover) on total fish density (Fig 9B). Finally, there were no direct or indirect effects of season on species richness and Shannon Index.

#### Covariance of species density

**Fig 10:**
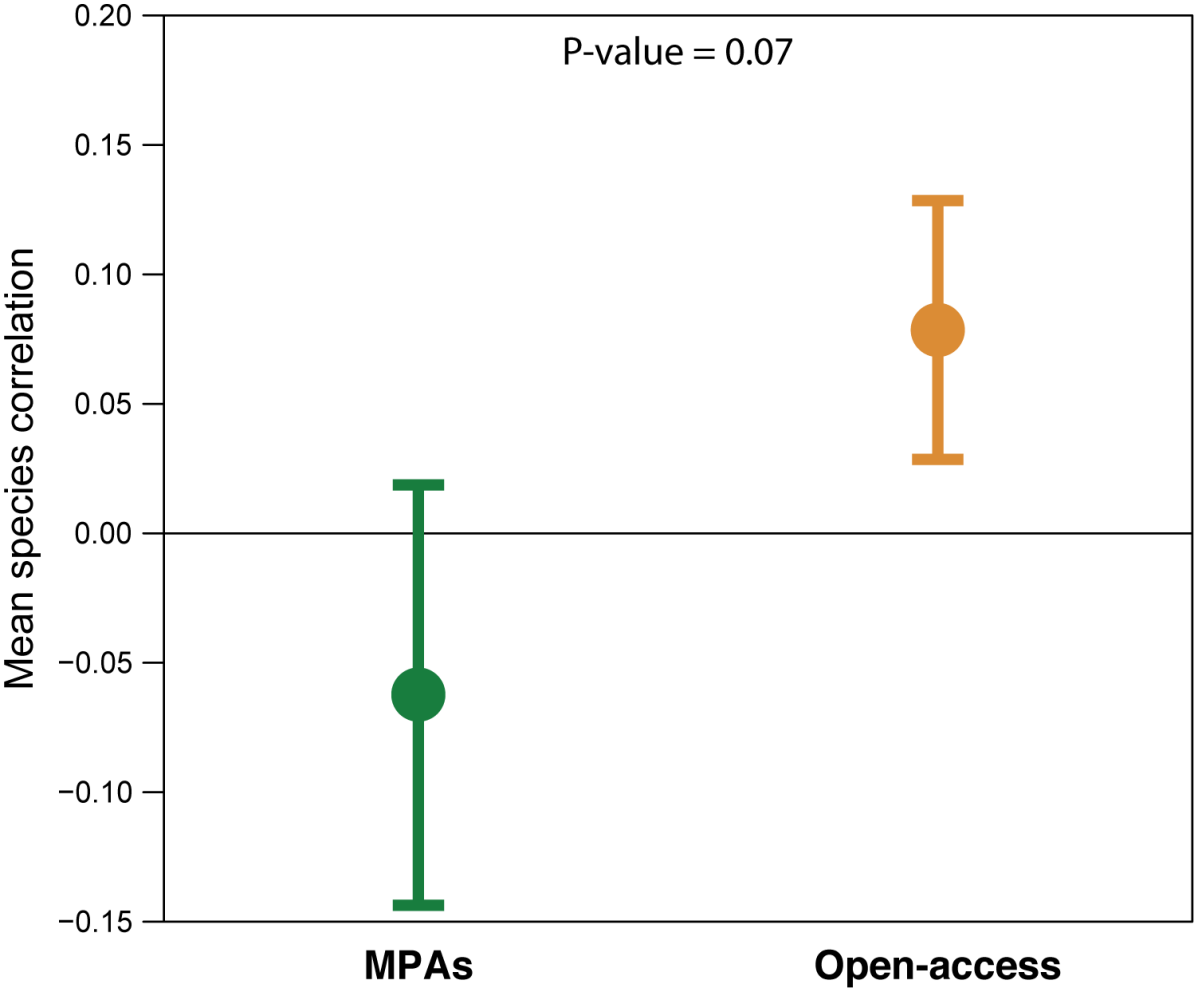
Mean correlations of species densities. Mean temporal correlations (± 1 SD) between all possible species pairs in MPAs (green) and open-access sites (orange).

Mean pair-wise correlation coefficients between densities of different fish species over time were close to zero in MPAs, while in open-access sites the average correlations were slightly positive. This difference was not significant (P = 0.07, Fig 10 and S1 Fig).

## Discussion

MPAs have generally been shown to increase the long-term temporal stability and resilience of marine communities to disturbances [9,15,74], but MPAs have also been shown to be at least as sensitive to disturbance as unprotected areas [7]. Here, we assessed the effects of two East African MPAs on the temporal stability of tropical seagrass-associated fish in relation to the yearly monsoon cycle. This is one of the most influential weather phenomena in the region, and likely to undergo considerable changes in the near future, in terms of temporal patterns and precipitation [27–30]. In short, we found that the effects of MPAs on the temporal stability of fish densities and community structure were weak. There were no effects on the densities and community structure of total and subadult fish. In the case of juvenile and adult fish, MPAs seemed to increase the temporal stability of the community structure, and juvenile fish densities were also more temporally stable within MPAs compared to open-access sites. Finally, species richness and diversity were not influenced by protection or seasonality.

MPAs did not increase fish density or diversity (species richness and Shannon Index); nor did they increase the temporal stability of these variables when compared to open-access sites. Moreover, an MPA effect on the stability of fish density was only apparent in the case of juvenile fish. These results partly contrast with those from many previous studies in the same region, demonstrating that MPAs generally increase fish density and diversity [57,75]. One potential reason for these differences could be that this is one of the first studies assessing MPA effects on seagrass-associated fish communities, while the large majority of other studies have focused on coral reef-associated fish (e.g. [75]). Seagrass fish communities are generally less diverse than those in coral reefs, and seagrass fish are typically more mobile and less sedentary than coral reef fish [35]. Both of these differences could reduce the effectiveness of MPAs. Another potential reason could be the relatively few sites included in this survey. Thus, any influence (or lack thereof) of a specific MPA was more heavily weighted in the overall results. Several previous studies have also questioned the effectiveness of MPAs as a management tool to conserve seagrass communities, both in East Africa [3] and in South-East Asia [4]. Therefore, our results suggest that at least for seagrass communities, these two MPAs do not seem to fulfil two of the main goals of marine conservation: to rebuild fish stocks and increase biodiversity.

When considering fish community structure, both juvenile and adult fish assemblage structures were temporally more stable (less seasonally variable) in the two MPAs than in the two open-access sites. Similar results have been observed for a range of MPAs when considering long-term interannual fluctuations [9,15–17]. One of the main suggested mechanisms is increased diversity within protected areas, which may promote stability of fish communities by increasing the likelihood of compensatory responses [11,12]. However, here, we found no effect of MPAs (or season) on fish diversity. There were no protection effects on the community structure of subadult fish, as well as the total fish community, potentially due to opposite seasonal changes for different age classes, resulting in an overall stable fish community. Below we discuss two potential mechanisms that could explain these results.

First, piecewise SEM revealed the presence of direct and indirect (seagrass-mediated) season-induced variability in fish densities in open-access sites, while no such seasonal differences were found in the MPAs. Seagrass cover is well-known to positively affect the density and diversity of fish assemblages [36–38], and seagrass cover can also change with season [40,41]. Indeed, our results indicated that seagrass cover was more variable over the seasons in open-access sites compared to MPAs, with indirect effects on fish abundance. The higher temporal variability in seagrass cover in open-access sites was not investigated in more detail, but could be caused by seasonal changes in human fishing activities that negatively affect seagrass cover (e.g. seine net fisheries) [76]. Regardless of the exact mechanism involved, these results suggest that MPAs, by reducing temporal variability in the cover of foundation species, can enhance the temporal stability of associated organisms like fish. Moreover, the lack of an indirect (seagrass-mediated) effect on fish densities within MPAs suggests that the temporal variability found in subadult and adult fish densities across both protected and open-access sites, may be at least partially driven by natural, seasonal reproduction, growth and/or migration patterns.

Piecewise SEM analyses also revealed a seasonality effect on adult and subadult fish densities in open-access sites but not within MPAs, which was not observed in previous analyses. This incongruence may be caused by the fact that separate piecewise SEM analyses were run for MPAs and open-access sites to be able to explore the interactive effects of management and seasonality on seagrass cover. This suggest that even though there is an overall seasonality effect on subadult and adult fish communities, this effect may be stronger in open-access sites, and easier to detect in the individual models.

In general, juvenile fish benefitted more from protection than other life stages, exhibiting higher stability within MPAs than subadult and adult fish. This pattern appeared to be mainly driven by parrotfish (Scaridae), which seemed to recruit to seagrass beds in MPAs throughout all three seasons (accounting for about 50% of the total juvenile fish community), while in open-access sites their presence was more seasonally variable (0 to 52%). The increased variability in the abundance of juvenile parrotfish in unprotected seagrass beds could be explained by differential recruitment and settlement patterns due to environmental conditions, such as oceanic currents, as well as more variable survival rates. Seagrass cover may offer protection from predation to juvenile fish [77], and thus, seasonal changes in seagrass cover may increase or decrease the risk of predation accordingly. As shown by our results, seagrass cover was more variable in open-access sites compared to MPAs, which could explain the increased stability of the juvenile fish community in MPAs. However, the increased stability of the juvenile community within MPAs was not shared by the other age classes. One potential explanation could be that fish migrations, either seasonal or ontogenetic, to/from other neighbouring habitats or areas, make the subadult and adult fish communities naturally less stable. Another possible explanation is that enforcement of MPAs may not be sufficient to avoid poaching, and thus fishing effects may still be detectable within MPAs, making subadult and adult fish communities less stable. Even though the Chumbe MPA has strong enforcement and poaching incidents are scarce [75], this might not be the case in the more recently established Mnemba MPA.

A second mechanism that could explain differences in the temporal stability of fish assemblages is how, and the extent to which, the densities of different species covary over time. Negative covariation over time may occur due to interspecific interactions or differential responses to changes in environmental conditions/disturbances, and cause compensatory changes that increase overall community stability [10,78]. Based on our data, pair-wise correlations of densities of the most common fish species in the MPAs displayed an average neutral (slightly negative) correlation coefficient, while the correlations in open-access sites were on average more positive. Although this difference was not significant, the strong trend suggests that temporal changes in the density of different species may be more synchronized in the two open-access sites, than in the MPAs. One potential reason for this difference could be the higher variability in seagrass cover in the open-access sites (see above). However, in the East African region, fishing is known to follow monsoonal seasonal patterns and to be highly unselective, with most fish species being caught regardless of size and species identity [50,52]. Thus, a pattern of positive covariance in species densities over time could also reflect a temporally changing effect of fishing; a pattern that would be apparent even if species diversity was high [78].

MPAs have been previously shown to buffer seagrass-associated fish assemblages from seasonal changes in the Mediterranean [19–21]. To our knowledge, this is the first study investigating such a buffer effect for tropical seagrass-associated fish communities, and in relation to the monsoon cycle. Our results suggest that this buffer effect may not be strong for these fish communities as a whole, but may benefit juvenile fish. A higher level of site replication may have strengthened these patterns and allowed us to dive even deeper into the potential mechanisms. We also recognise that the proposed mechanisms may be acting in conjunction, and that other mechanisms not discussed here may also be involved. For example, increased predation rates inside MPAs could limit variability in prey organisms [17] and reduce natural fluctuations in recruitment [19]. Moreover, the sites where MPAs have been established could be naturally more stable than the open-access sites, which would result in patterns similar to the ones observed in the present study.

In conclusion, our findings suggest that while these MPAs increase the temporal stability of certain groups, particularly juvenile fish, by buffering both direct and habitat-mediated (indirect) effects of monsoon seasonality, this effect may be minimal when considering the whole seagrass fish community. From a scientific perspective, these results emphasize the need to separate fish communities into age groups to accurately identify effects. From a management perspective, our results suggest that these MPAs may not have the capacity to fully buffer the effects of changing monsoon patterns under future scenarios. This strongly emphasizes the need for more studies that critically assess the role of MPAs to facilitate the maintenance of seagrass ecosystems and their associated services, particularly in light of global environmental change.

## Supporting information

S1 Appendix Reference site selection. (PDF)

S2 Appendix Piecewise structural equation modelling. (PDF)

S1 Fig Slopes of correlation between species pairs. Slopes of correlation between all possible species pairs in (A) MPAs and (B) open-access sites. Slopes are coloured by sign and management level (Green: negative + MPA; Blue: positive + MPA; Red: negative + Open-access area; Orange: positive + Open-access area). Black lines represent the mean slopes of correlation.
(TIF)
