# Supplementary material for: Marine protected areas increase temporal stability of community structure, but not density or diversity, of tropical seagrass fish communities
Source: PLoS One. 2017 Aug 30;12(8):e0183999. doi: 10.1371/journal.pone.0183999 (PMC5576671; doi:10.1371/journal.pone.0183999)
Supplement: S1 Appendix — (PDF) [file pone.0183999.s001.pdf]

S1 Appendix. Reference site selection.

Reference sites were selected to have as similar environmental conditions to each of the marine protected areas (MPAs) as possible. Given the off-shore location of both MPAs, reference sites were also chosen from locations off-shore from Unguja island. From the few available locations, the reference sites were then chosen based on the presence of *Thalassodendron ciliatum* beds and their similarity to their respective MPA sites in terms of wave exposure and distance to coral reefs, factors known to affect seagrass-associated fish assemblages [1-3].

To assess the possible differences between sites, we measured the exposure and distance to coral reef from each of the four sampling sites (Table 1). Distance to the nearest coral reef from each sampling site was estimated using Google Earth pro (version 7.1) from satellite images taken from 2014 to 2015. Exposure was calculated in terms of effective fetch (in km). The distance from each site to land was measured for 14 different directions (in 6° intervals to either side from the perpendicular to the shore), and effective fetch was calculated as [4]:

$$\text{Effective fetch} = \frac{\sum x_i \cos y_i}{\sum \cos y_i}$$

where yi is the angle of a given direction and xi is the distance to land in that direction.

We then compared the measured variables between MPAs and fished sites using a two-sample paired t-test (`'t.test'` in `{stats}`) [5]. Differences between MPAs and reference sites were not significant for either exposure or distance to coral reef (P = 0.23 and P = 0.38, respectively).

Table 1. Sampling site characteristics.

Site	Protection	Exposure (km)	Distance to reef (m)
Changuu	None	38.8	54
Chumbe	MPA	27.2	45
Mnemba MPA	MPA	13.0	583
Mnemba reef	None	7.9	630

References

1. Gullström M, Bodin M, Nilsson PG, Öhman MC. Seagrass structural complexity and landscape configuration as determinants of tropical fish assemblage composition. Mar Ecol Prog Ser. Inter-Research; 2008;363: 241–255. doi:10.3354/meps07427
2. Alonso Aller E, Gullström M, Eveleens Maarse FKJ, Gren M, Nordlund LM, Jiddawi N, et al. Single and joint effects of regional- and local-scale variables on tropical seagrass fish assemblages. Mar Biol. Springer Berlin Heidelberg; 2014;161: 2395–2405. doi:10.1007/s00227-014-2514-7
3. Turner SJ, Hewitt JE, Wilkinson MR, Morrisey DJ, Thrush SF, Cummings VJ, et al. Seagrass patches and landscapes: the influence of wind-wave dynamics and hierarchical arrangements of spatial structure on macrofaunal seagrass communities. Estuaries. Coastal and Estuarine Research Federation; 1999;22: 1016–1032. doi:10.2307/1353080
4. Håkanson L, Jansson M. Principles of Lake Sedimentology. Berlin; New York: Springer-Verlag; 1983.
5. R Core Team. R: a language and environment for statistical computing. 3rd ed. Vienna, Austria: R Foundation for Statistical Computing; 2016.
