# Supplementary material for: Marine protected areas increase temporal stability of community structure, but not density or diversity, of tropical seagrass fish communities
Source: PLoS One. 2017 Aug 30;12(8):e0183999. doi: 10.1371/journal.pone.0183999 (PMC5576671; doi:10.1371/journal.pone.0183999)
Supplement: S2 Appendix — (PDF) [file pone.0183999.s002.pdf]

S2 Appendix. Piecewise Structural Equation Modelling.

We fitted an initial general model with two components: one with total fish density as response variable and seagrass cover and an interaction between seasonality and management as predictors; and the second one with seagrass cover as a response, and an interaction between seasonality and management as predictors. The same general model was also fitted for each of the other univariate response variables (juvenile, sub-adult and adult fish densities, and species diversity and Shannon Index). Each component was fitted as a linear mixed effects model, with site as a random factor nested within each level of management. The variable season, being a factor with three levels, was modelled as a composite variable [1]. Models were fitted with the R package `{piecewiseSEM}` [2]. Significance levels were set at $\alpha = 0.05$, and each transect was used as a replicate ($N = 111$).

The results showed that there was a significant effect of the interaction between seasonality and management for all fish densities response variables (P-value < 0.05). However, these results did not allow us to interpret the interaction between management and season further. We then fitted individual models for each management level (MPAs and fished areas) to better understand the interactive effects of the two factors (see main text). No significant direct or indirect effects of management or seasonality were found on species richness and Shannon Index.

References

1. Grace JB. Structural Equation Modeling and Natural Systems. Cambridge, UK: Cambridge University Press; 2006.
2. Lefcheck JS. piecewiseSEM: Piecewise structural equation modeling in R for ecology, evolution, and systematics. Methods Ecol Evol. 2015: 573–579. doi:10.1111/2041-210X.12512
