# Supplementary figures and images for: Marine protected areas increase temporal stability of community structure, but not density or diversity, of tropical seagrass fish communities
Source: PLoS One. 2017 Aug 30;12(8):e0183999. doi: 10.1371/journal.pone.0183999 (PMC5576671; doi:10.1371/journal.pone.0183999)

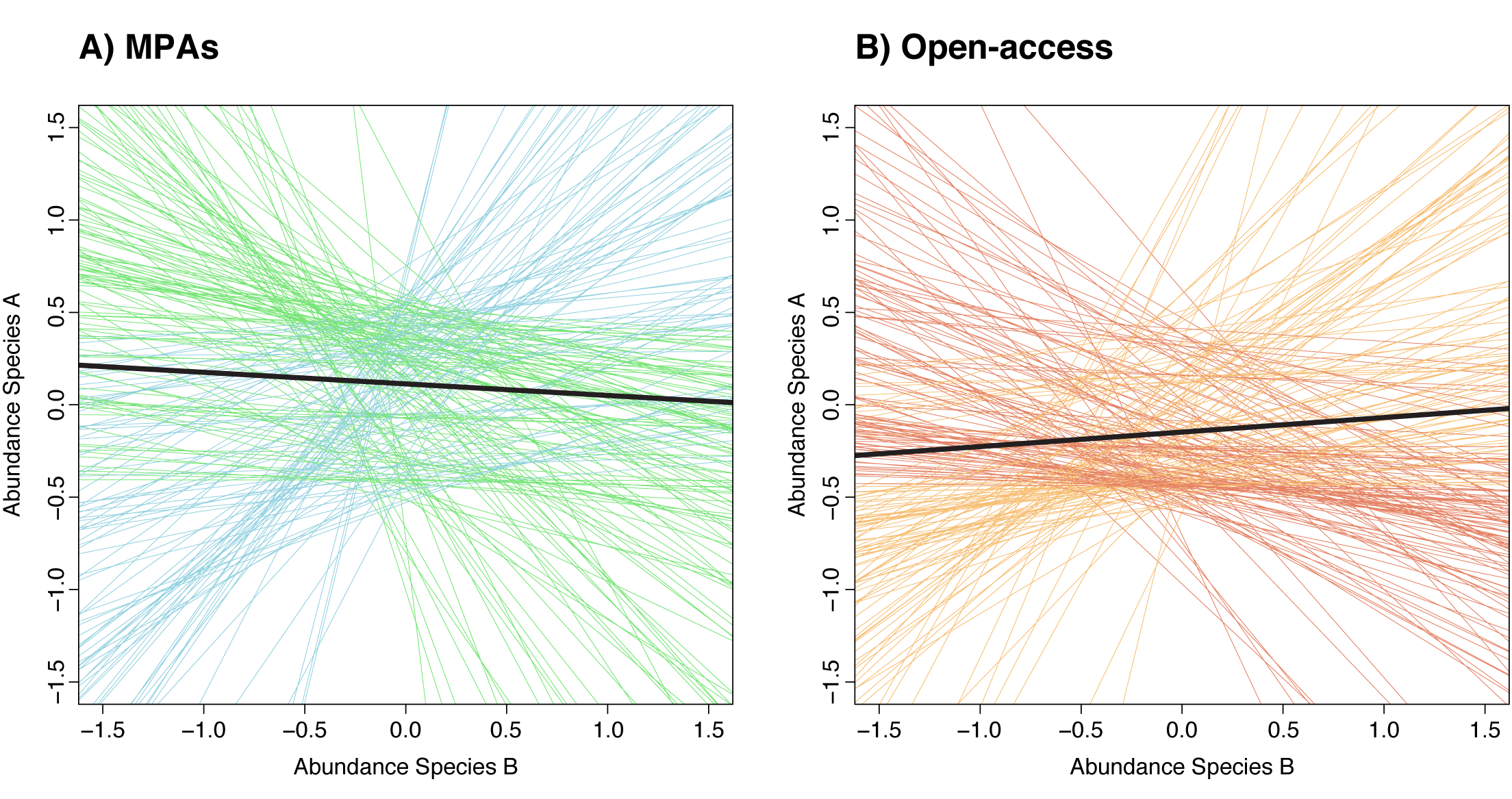

Supplement: S1 Fig — Slopes of correlation between all possible species pairs in (A) MPAs and (B) open-access sites. Slopes are coloured by sign and management level (Green: negative + MPA; Blue: positive + MPA; Red: negative + Open-access area; Orange: positive + Open-access area). Black lines represent the mean slopes of correlation. (TIF) [file pone.0183999.s003.tif]
